# Postoperative Analgesic Effects of Pre-emptive Intravenous Paracetamol Versus Ibuprofen in Laparoscopic Cholecystectomy: A Randomized, Double-Blind Study

**DOI:** 10.7759/cureus.113753

**Published:** 2026-07-31

**Authors:** Ashwani Raj, Arvind Kumar, Shashank Dhiraj, Shashi Kant, Sanjay Kumar, Rajesh Kumar

**Affiliations:** 1 Anaesthesia, Indira Gandhi Institute of Medical Sciences, Patna, Patna, IND; 2 Anaesthesiology, Indira Gandhi Institute of Medical Sciences, Patna, Patna, IND; 3 Anaesthesia, All India Institute of Medical Sciences (AIIMS) Patna, Patna, IND; 4 Anaesthesiology, All India Institute of Medical Sciences (AIIMS) Patna, Patna, IND

**Keywords:** cyclooxygenase, food and drug administration, mean arterial pressure, nonsteroidal anti-inflammatory drugs, randomized controlled trials, visual analogue score

## Abstract

Introduction

Effective postoperative pain control is essential for enhanced recovery and improved patient satisfaction following laparoscopic cholecystectomy. Pre-emptive administration of non-opioid analgesics, such as intravenous paracetamol and ibuprofen, has been incorporated into multimodal analgesic strategies to reduce postoperative pain and opioid consumption. This study compared the analgesic efficacy and safety of pre-emptive intravenous paracetamol and intravenous ibuprofen in patients undergoing elective laparoscopic cholecystectomy.

Objective

The primary objective of this study was to compare postoperative pain intensity between pre-emptive intravenous paracetamol and intravenous ibuprofen using the Visual Analogue Scale (VAS) at the end of surgery and at 2, 6, 12, 18, and 24 hours postoperatively. Secondary objectives included comparison of perioperative haemodynamic parameters (heart rate, mean arterial pressure, and peripheral oxygen saturation) and assessment of drug-related adverse effects, including postoperative nausea and vomiting.

Methodology

This prospective, randomized, double-blind, parallel-group comparative study was conducted in the Department of Anaesthesiology and Critical Care, Indira Gandhi Institute of Medical Sciences (IGIMS), Patna, India, between August 2023 and January 2025. Ninety patients aged 18-60 years with American Society of Anesthesiologists (ASA) physical status I or II undergoing elective laparoscopic cholecystectomy under general anaesthesia were randomly allocated into two equal groups. Group P received intravenous paracetamol 2 g, while Group I received intravenous ibuprofen 800 mg, each diluted in 200 mL normal saline and infused over 30 minutes before induction of anaesthesia. Postoperative pain was assessed using the Visual Analogue Scale, and haemodynamic variables and adverse events were recorded for 24 hours.

Results

Baseline demographic and clinical characteristics were comparable between the two groups. Postoperative VAS scores improved over time in both groups, with a significantly greater reduction in pain scores in the ibuprofen group than in the paracetamol group (p < 0.001). Heart rate and other haemodynamic parameters remained comparable between groups throughout the study, with no significant difference in overall heart rate trends (p = 0.060). Although intravenous ibuprofen provided superior analgesia, it was associated with a higher incidence of postoperative nausea and vomiting.

Conclusion

Pre-emptive intravenous ibuprofen provided superior postoperative analgesia compared with intravenous paracetamol following laparoscopic cholecystectomy. However, because ibuprofen was associated with a higher incidence of adverse effects, it should be selected with attention to patient characteristics and clinical risk factors.

## Introduction

Laparoscopic cholecystectomy is the preferred surgical treatment for symptomatic gallstone disease and is considered the standard of care because of its minimally invasive approach, shorter hospital stay, faster recovery, lower postoperative morbidity, and improved patient satisfaction compared with open cholecystectomy. Despite these advantages, postoperative pain remains a common clinical challenge, particularly during the first 24 hours after surgery. The pain is multifactorial in origin and results from trocar-site incisions, gallbladder dissection, peritoneal stretching caused by pneumoperitoneum, diaphragmatic irritation, and residual carbon dioxide within the abdominal cavity. Inadequately controlled postoperative pain delays mobilisation, impairs recovery, prolongs hospitalisation, and adversely affects patient satisfaction and quality of recovery. Therefore, effective postoperative analgesia is a key component of perioperative care and Enhanced Recovery After Surgery (ERAS) protocols [1-3].

Opioids have traditionally been the cornerstone of postoperative pain management because of their potent analgesic effects. However, opioid administration is frequently associated with adverse effects such as postoperative nausea and vomiting, respiratory depression, excessive sedation, ileus, urinary retention, delayed mobilisation, and the potential for tolerance and dependence. These limitations have encouraged the adoption of opioid-sparing multimodal analgesic strategies, which combine analgesics with different mechanisms of action to improve pain control while minimizing opioid consumption and opioid-related adverse effects [1-4].

Among the non-opioid analgesics used in multimodal analgesia, intravenous paracetamol and intravenous ibuprofen are widely recommended because of their rapid onset of action, proven analgesic efficacy, and favourable safety profiles. Intravenous paracetamol exerts its analgesic effect primarily through central inhibition of prostaglandin synthesis and modulation of serotonergic pathways, while having minimal effects on platelet function and renal perfusion. Intravenous ibuprofen, a non-selective cyclooxygenase (COX)-1 and COX-2 inhibitor, provides both analgesic and anti-inflammatory effects by suppressing peripheral prostaglandin synthesis, thereby reducing postoperative pain and inflammatory responses following surgery [1,3-6].

Recent systematic reviews and meta-analyses have demonstrated that perioperative intravenous ibuprofen significantly reduces postoperative pain intensity, opioid consumption, and postoperative fever while maintaining an acceptable safety profile. Likewise, perioperative intravenous acetaminophen (paracetamol) has been shown to provide effective analgesia and contribute to opioid-sparing multimodal analgesia. Current Procedure-Specific Postoperative Pain Management (PROSPECT) recommendations also advocate the routine use of paracetamol and non-steroidal anti-inflammatory drugs (NSAIDs), unless contraindicated, as part of multimodal postoperative pain management following laparoscopic cholecystectomy [1,2,4-6].

Although both intravenous paracetamol and intravenous ibuprofen are recommended as components of multimodal analgesia, direct comparative evidence evaluating these two agents in patients undergoing laparoscopic cholecystectomy remains limited. Most published studies have compared either drug with placebo or opioid-based analgesic regimens rather than directly comparing their relative efficacy and safety. Consequently, uncertainty remains regarding the optimal non-opioid analgesic for routine perioperative pain management after laparoscopic cholecystectomy [2,4-6].

Therefore, the present study was undertaken to compare the analgesic efficacy and safety of pre-emptive intravenous paracetamol and intravenous ibuprofen in patients undergoing elective laparoscopic cholecystectomy. The primary objective was to compare postoperative pain intensity using the Visual Analogue Scale (VAS) [7], while secondary outcomes included rescue analgesic requirement, haemodynamic changes, postoperative nausea and vomiting, adverse effects, and overall quality of postoperative analgesia during the first 24 postoperative hours.

## Materials and methods

Study design and setting

This prospective, randomized, double-blind, parallel-group comparative study was conducted in the Department of Anaesthesiology and Critical Care at Indira Gandhi Institute of Medical Sciences (IGIMS), Patna, Patna, India (approval no. 944/IEC/IGIMS/2023, date: 10-04-2023), from August 2023 to January 2025. The study protocol was approved by the Institutional Ethics Committee (Reference No. REF/2023/05/067962) and prospectively registered with the Clinical Trials Registry-India (CTRI No. CTRI/2023/06/054441). Written informed consent was obtained from all participants prior to enrolment.

Study population

Patients aged 18-60 years with American Society of Anesthesiologists (ASA) Physical Status I or II, according to the American Society of Anesthesiologists Physical Status Classification System [8], who were scheduled to undergo elective laparoscopic cholecystectomy under general anaesthesia were screened for eligibility. Patients were excluded if they declined participation, required emergency surgery, had a known hypersensitivity to paracetamol or ibuprofen, were pregnant or breastfeeding, had a body mass index >35 kg/m², a Modified Mallampati Score (MPS) ≥3 [9], or had renal failure, chronic liver disease, endocrine disorders, rheumatic disease, malignancy, coagulation abnormalities, or were receiving anticoagulant therapy. The indication for laparoscopic cholecystectomy was determined by the treating surgical team according to established clinical practice guidelines [1].

Randomisation and blinding

Eligible participants were randomly assigned in a 1:1 ratio to either the intravenous paracetamol group (Group P) or the intravenous ibuprofen group (Group I) using a computer-generated randomization sequence. Allocation concealment was ensured using sequentially numbered, opaque, sealed envelopes. An anaesthesiologist who was not involved in intraoperative management or postoperative assessment prepared the study medications, thereby maintaining blinding of the patients, surgeons, attending anaesthesiologists, investigators, and outcome assessors throughout the study.

Study intervention

Patients allocated to Group P received intravenous paracetamol 2 g diluted in 200 mL of normal saline administered over 30 minutes before induction of anaesthesia. Patients allocated to Group I received intravenous ibuprofen 800 mg diluted in 200 mL of normal saline infused over the same duration before induction. The study drug doses and timing of administration were based on previous studies evaluating pre-emptive intravenous analgesia in patients undergoing laparoscopic cholecystectomy [10].

Anaesthetic technique

All patients underwent a routine pre-anaesthetic evaluation and observed a fasting period of at least six hours before surgery. Standard intraoperative monitoring included electrocardiography, non-invasive blood pressure, pulse oximetry, capnography, and Bispectral Index (BIS) monitoring (BIS LoC 2 Channel monitor, Covidien, Dublin, Ireland). General anaesthesia was induced with intravenous fentanyl (2 μg/kg), propofol (2 mg/kg), and atracurium (0.5 mg/kg), followed by endotracheal intubation. Mechanical ventilation was adjusted to maintain an end-tidal carbon dioxide concentration between 35 and 40 mmHg. Anaesthesia was maintained with oxygen, nitrous oxide, and isoflurane while maintaining the BIS value between 45 and 55. Intravenous ondansetron 4 mg was administered approximately 30 minutes before skin closure for prophylaxis against postoperative nausea and vomiting. Residual neuromuscular blockade was reversed with intravenous neostigmine (50 μg/kg) and glycopyrrolate (20 μg/kg), followed by tracheal extubation after fulfilment of standard extubation criteria.

Outcome measures

The primary outcome was postoperative pain intensity assessed using a validated 10-cm Visual Analogue Scale (VAS), where 0 represented no pain and 10 represented the worst imaginable pain [7]. Pain scores were recorded immediately after surgery (0 hour) and at 2, 6, 12, 18, and 24 hours postoperatively. Secondary outcome measures included heart rate, systolic blood pressure, diastolic blood pressure, mean arterial pressure, peripheral oxygen saturation, time to first rescue analgesia, total rescue analgesic requirement during the first 24 postoperative hours, incidence of postoperative nausea and vomiting, and any drug-related adverse events. Haemodynamic variables were recorded before induction, at predefined intraoperative time points, and throughout the postoperative observation period.

Postoperative analgesia

Following surgery, all patients were transferred to the post-anaesthesia care unit for routine monitoring. No scheduled postoperative analgesic was administered immediately after surgery. Rescue analgesia consisted of intravenous tramadol (2 mg/kg) whenever the VAS score was ≥7. The time to first rescue analgesia, cumulative tramadol consumption during the first 24 hours, and adverse events including nausea, vomiting, headache, pruritus, and other complications were documented.

Sample size and statistical analysis

Sample size estimation was performed using the method described by Dell et al. [11]. Assuming a 90% statistical power and a 95% confidence level, a minimum sample size of 41 patients per group was required. To compensate for potential dropouts and improve study precision, 45 patients were enrolled in each group. Data were entered into Microsoft Excel (Microsoft Corporation, Redmond, WA, USA) and analyzed using IBM SPSS Statistics for Windows, Version 23.0 (IBM Corp., Armonk, NY, USA). Continuous variables are presented as mean ± standard deviation or median (interquartile range), depending on the distribution of the data, while categorical variables are expressed as frequencies and percentages. Normality was assessed before analysis. Continuous variables were compared using the independent-samples t-test for normally distributed data or the Mann-Whitney U test for non-normally distributed data. Categorical variables were analyzed using the Chi-square test or Fisher's exact test, as appropriate.

For repeated postoperative measurements, non-parametric statistical methods were applied because the data were not normally distributed. The Friedman test was used to evaluate changes within each group over time, the Wilcoxon-Mann-Whitney test was used to compare the two groups at each time point, and generalized estimating equations (GEE) were used to assess differences in the longitudinal trends between groups. A two-sided p value <0.05 was considered statistically significant.

## Results

**Figure 1:**
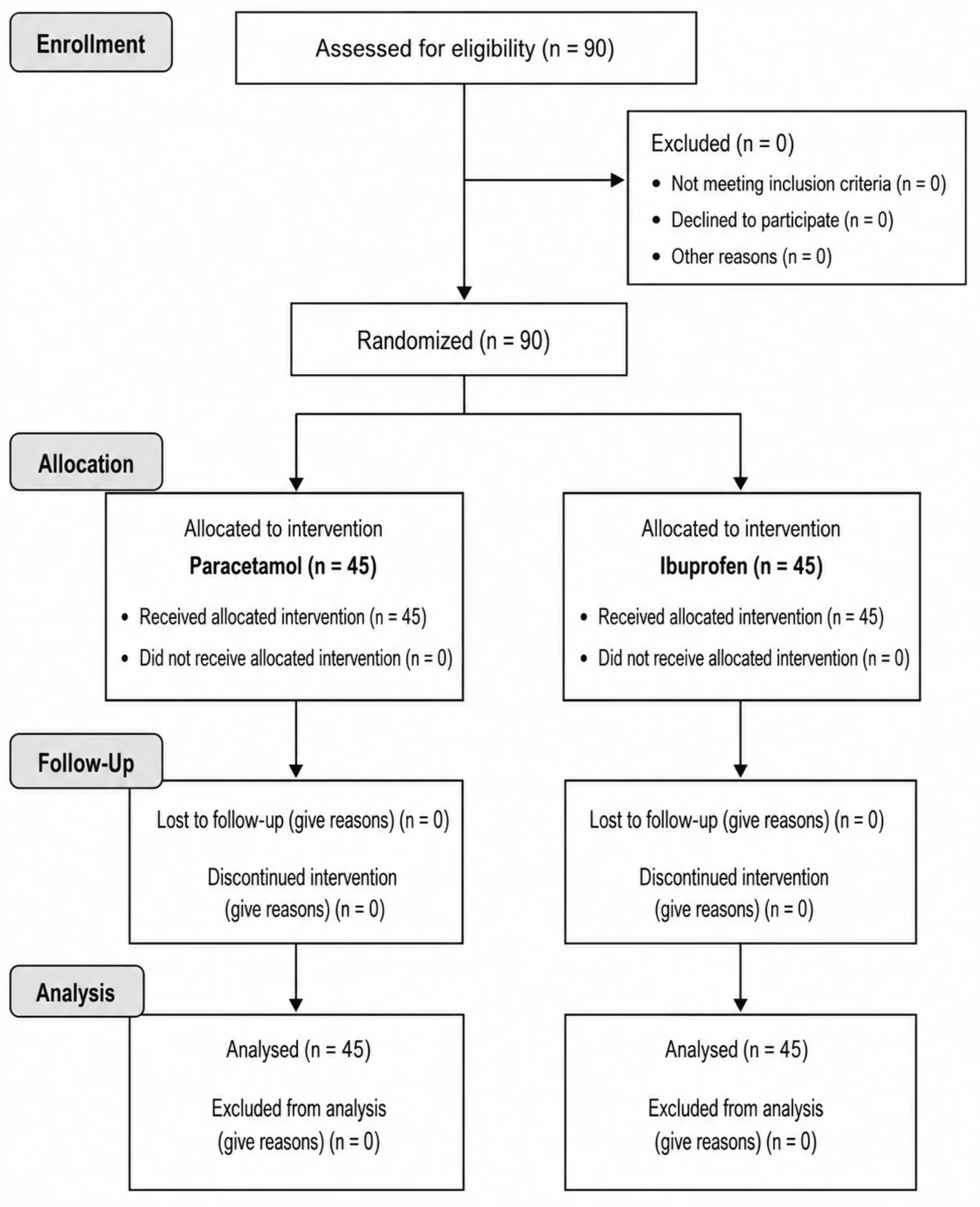
CONSORT Flow Diagram Showing Participant Enrolment, Randomization, Allocation, Follow-Up, and Analysis of the Study Population CONSORT: Consolidated Standards of Reporting Trials. CONSORT flow diagram illustrating participant screening, randomization, allocation to the intravenous paracetamol (Group P) and intravenous ibuprofen (Group I) groups, follow-up, and final analysis. A total of 90 participants were randomized, with 45 participants allocated to each group. All participants received the allocated intervention, completed follow-up, and were included in the final analysis.

A total of 90 patients were assessed for eligibility and enrolled in the study. All eligible patients were randomized equally into two groups: Group P (intravenous paracetamol, n = 45) and Group I (intravenous ibuprofen, n = 45). All randomized patients completed the study protocol and were included in the final analysis. No patient was lost to follow-up or excluded from the analysis (Figure 1).

There were no significant differences in baseline demographic or clinical characteristics between the two groups, confirming successful randomisation. Continuous variables, including age, were expressed as mean ± standard deviation (SD) and compared using the independent-samples t-test, whereas categorical variables, including sex, American Society of Anesthesiologists (ASA) physical status, and Modified Mallampati Score (MPS) [9], were compared using the Chi-square test. The groups were comparable with respect to age, sex distribution, ASA physical status, and MPS (Table 1).

**Table 1:** Demographic Characteristics of the Patients in Group I and Group P Data are presented as mean ± standard deviation (SD) or number (%). SD: standard deviation, n: sample size, ASA: American Society of Anesthesiologists physical status classification, MPS: Modified Mallampati Score, p-value: probability value (p < 0.05 is considered statistically significant), Group P: intravenous paracetamol group, Group I: intravenous ibuprofen group. The statistical tests used were the independent-samples t-test¹ and Pearson Chi-square test².

Parameters	Group I (n = 45)	Group P (n = 45)	p value
Age (Years)	37.22 ± 12.67	37.42 ± 12.15	0.923¹
Age	-	-	-
<20 Years	3 (6.7%)	1 (2.2%)	0.490²
20-29 Years	14 (31.1%)	12 (26.7%)	
30-39 Years	8 (17.8%)	13 (28.9%)	
40-49 Years	11 (24.4%)	12 (26.7%)	
50-59 Years	8 (17.8%)	4 (8.9%)	
60-69 Years	1 (2.2%)	3 (6.7%)	
Gender	-	-	-
Male	18 (40.0%)	18 (40.0%)	1.000^2^
Female	27 (60.0%)	27 (60.0%)	
ASA Grade	-	-	-
I	39 (86.7%)	40 (88.9%)	0.748^2^
II	6 (13.3%)	5 (11.1%)	
MPS	-	-	-
Score 1	13 (28.9%)	8 (17.8%)	0.460^2^
Score 2	26 (57.8%)	30 (66.7%)	
Score 3	6 (13.3%)	7 (15.6%)	

Postoperative pain scores

Changes in postoperative Visual Analogue Scale (VAS) scores over time were analyzed within each group using the Friedman test and between groups using generalized estimating equations (GEE). The Mann-Whitney U test was used for intergroup comparisons at each postoperative time point. In Group I, the mean VAS score increased from 1.51 immediately after surgery to a peak of 4.58 at six hours, then declined to 1.73 at 24 hours. This change over time was statistically significant (Friedman test: χ² = 139.2, p < 0.001).

In Group P, the mean VAS score increased from 2.51 immediately after surgery to 4.58 at two hours and then gradually decreased to 2.29 at 24 hours, showing a significant change over time (Friedman test: χ² = 156.3, p < 0.001). The generalized estimating equations model showed a significant difference in postoperative pain trajectories between the two groups (p < 0.001). Intergroup comparisons showed lower VAS scores in the ibuprofen group at 0, 2, 6, 12, 18, and 24 hours after surgery (Table 2).

**Table 2:** Comparison in Groups P and I in Terms of Change in VAS Over Time VAS: Visual Analogue Scale, SD: standard deviation, n: sample size, p-value: probability value (p < 0.05 is considered statistically significant), Group P: intravenous paracetamol group, Group I: intravenous ibuprofen group. The statistical test used for comparison between groups at each time point was the Wilcoxon-Mann-Whitney test, the Friedman test was used for within-group comparisons over time, and Generalized Estimating Equations (GEE) were used for overall comparison of longitudinal trends between the groups.

VAS	Group		p value for comparison of the two groups at each of the timepoints (Wilcoxon-Mann-Whitney Test)
	I	P	
	Mean (SD)	Mean (SD)	
0 Hours	1.51 (0.69)	2.51 (0.79)	<0.001
2 Hours	3.58 (1.01)	4.58 (0.75)	<0.001
6 Hours	4.58 (2.09)	3.20 (0.79)	<0.001
12 Hours	3.13 (0.94)	3.69 (0.67)	0.004
18 Hours	2.42 (0.78)	2.87 (0.55)	0.005
24 Hours	1.73 (0.54)	2.29 (0.59)	<0.001
p value for change in VAS over time within each group (Friedman Test)	<0.001	<0.001	-
Overall p value for comparison of change in VAS over time between the two groups (Generalized Estimating Equations)	<0.001		

Heart rate

Heart rate changed significantly over time within both groups. In Group I, the mean heart rate increased from 93.49 beats/min before induction to 94.73 beats/min at 2 minutes after induction and then decreased to 83.13 beats/min at 24 hours (Friedman test: χ² = 100.0, p < 0.001). In Group P, the mean heart rate increased from 89.29 beats/min before induction to 98.29 beats/min at 30 minutes intraoperatively and then decreased to 81.29 beats/min at 24 hours (Friedman test: χ² = 72.3, p < 0.001). However, comparison of heart rate trends between the two groups using the generalized estimating equations model demonstrated no statistically significant difference (p = 0.060) (Table 3).

**Table 3:** Comparison in Group P and I in Terms of Changes in Heart Rate Over Time BPM: beats per minute, SD: standard deviation, n: sample size, p-value: probability value (p < 0.05 is considered statistically significant), Group P: intravenous paracetamol group, Group I: intravenous ibuprofen group. The statistical test used for comparison between groups at each time point was the Wilcoxon-Mann-Whitney test, the Friedman test was used for within-group comparisons over time, and Generalized Estimating Equations (GEE) were used for overall comparison of longitudinal trends between the groups.

Heart Rate (BPM)	Group		p value for comparison of the two groups at each of the timepoints (Wilcoxon-Mann-Whitney Test)
	I	P	
	Mean (SD)	Mean (SD)	
Pre-Induction	93.49 (12.70)	89.29 (12.74)	0.116
2 Minutes	94.73 (12.82)	87.51 (13.24)	0.022
10 Minutes	93.91 (12.45)	86.13 (11.49)	0.004
20 Minutes	91.93 (10.28)	98.16 (92.71)	0.005
30 Minutes	92.09 (10.44)	98.29 (92.61)	0.001
40 Minutes	94.07 (17.99)	85.89 (11.86)	0.020
1 Hour	92.84 (17.30)	88.44 (9.32)	0.348
2 Hours	90.76 (12.03)	88.29 (8.04)	0.528
6 Hours	87.93 (11.33)	86.31 (7.75)	0.596
12 Hours	85.84 (10.53)	83.91 (6.08)	0.780
18 Hours	84.91 (10.96)	82.58 (6.44)	0.659
24 Hours	83.13 (10.01)	81.29 (5.82)	0.852
p value for change in heart rate (BPM) over time within each group (Friedman Test)	<0.001	<0.001	-
Overall p value for comparison of change in heart rate (BPM) over time between the two groups (Generalized Estimating Equations)	0.060		

Mean arterial pressure

Mean arterial pressure (MAP) remained stable throughout the study period in both groups. In Group I, MAP decreased from 94.67 mmHg before induction to 89.71 mmHg at 24 hours; however, this change was not statistically significant (Friedman test: χ² = 18.6, p = 0.069). In Group P, MAP increased slightly from 94.29 mmHg before induction to 94.69 mmHg at 2 hours before decreasing to 89.49 mmHg at 24 hours, and this change reached statistical significance (Friedman test: χ² = 50.7, p < 0.001). Despite these within-group changes, longitudinal comparison between groups using the generalized estimating equations model showed no significant difference in MAP over time (p = 0.349) (Table 4).

**Table 4:** Comparison in Group P and I in Terms of Change in MAP Over Time MAP: mean arterial pressure, SD: standard deviation, n: sample size, p-value: probability value (p < 0.05 is considered statistically significant), Group P: intravenous paracetamol group, Group I: intravenous ibuprofen group. The statistical test used for comparison between groups at each time point was the Wilcoxon-Mann-Whitney test, the Friedman test was used for within-group comparisons over time, and Generalized Estimating Equations (GEE) were used for overall comparison of longitudinal trends between the groups.

MAP (mmHg)	Group		p value for comparison of the two groups at each of the timepoints (Wilcoxon-Mann-Whitney Test)
	I	P	
	Mean (SD)	Mean (SD)	
Pre-Induction	94.67 (14.52)	94.29 (8.92)	0.984
2 Minutes	92.09 (14.62)	91.27 (9.35)	0.561
10 Minutes	91.78 (14.63)	87.40 (9.14)	0.051
20 Minutes	90.93 (11.52)	87.73 (9.04)	0.133
30 Minutes	91.04 (9.87)	90.42 (9.83)	0.628
40 Minutes	91.76 (8.80)	91.18 (9.41)	0.780
1 Hour	91.44 (9.03)	92.60 (7.66)	0.566
2 Hours	92.53 (7.54)	94.69 (6.49)	0.178
6 Hours	91.67 (5.85)	92.56 (4.15)	0.400
12 Hours	90.84 (5.63)	91.16 (3.90)	0.725
18 Hours	90.47 (5.96)	90.29 (4.09)	0.890
24 Hours	89.71 (6.01)	89.49 (4.70)	0.802
p value for change in MAP (mmHg) over time within each group (Friedman Test)	0.069	<0.001	-
Overall p value for comparison of change in MAP (mmHg) over time between the two groups (Generalized Estimating Equations)	0.349		

## Discussion

The present randomized comparative study evaluated the analgesic efficacy and safety of pre-emptive intravenous paracetamol and intravenous ibuprofen in patients undergoing elective laparoscopic cholecystectomy. Effective postoperative pain management is a key component of enhanced recovery pathways because inadequate analgesia delays ambulation, prolongs hospitalisation, increases postoperative morbidity, and adversely affects patient satisfaction. In the present study, patients receiving intravenous ibuprofen experienced significantly lower postoperative pain scores during the first 24 hours than those receiving intravenous paracetamol. However, this improved analgesia was accompanied by a higher incidence of postoperative nausea and vomiting, whereas intravenous paracetamol demonstrated a more favourable safety profile and greater haemodynamic stability [6,10,12,13].

Pain after laparoscopic cholecystectomy is multifactorial, arising from trocar-site incisions, visceral manipulation, diaphragmatic irritation caused by carbon dioxide pneumoperitoneum, and the inflammatory response associated with tissue injury. Contemporary multimodal analgesic strategies emphasise the use of non-opioid analgesics to improve postoperative pain control while minimizing opioid exposure. Recent systematic reviews and randomized controlled trials have demonstrated that intravenous ibuprofen is an effective component of multimodal analgesia for reducing postoperative pain following minimally invasive surgery [6,10,13,14].

The significantly lower postoperative Visual Analogue Scale (VAS) scores observed in the ibuprofen group are consistent with its pharmacological action. Ibuprofen inhibits both cyclooxygenase-1 and cyclooxygenase-2 enzymes, thereby suppressing peripheral prostaglandin synthesis and reducing inflammation-induced nociceptor sensitisation. In contrast, paracetamol exerts predominantly central analgesic effects with relatively limited peripheral anti-inflammatory activity. These pharmacological differences may explain the superior postoperative analgesia observed with intravenous ibuprofen in the present study [6,10].

Our findings are consistent with previous randomized controlled trials. Kim et al. demonstrated that perioperative intravenous ibuprofen provided superior postoperative analgesia and reduced opioid consumption compared with acetaminophen after general anaesthesia [6]. Similarly, Mohammadian Erdi et al. reported improved postoperative pain control with intravenous ibuprofen compared with acetaminophen while observing a slightly higher incidence of postoperative nausea and vomiting [10]. Tariq et al. also demonstrated significantly lower postoperative pain scores with intravenous ibuprofen than with intravenous paracetamol in patients undergoing laparoscopic cholecystectomy, findings that closely parallel those of the present study [12]. Likewise, Bulut et al. reported superior analgesic efficacy of intravenous ibuprofen following laparoscopic cholecystectomy [13].

Although postoperative opioid consumption was not the primary endpoint of our study, patients receiving intravenous ibuprofen required rescue analgesia less frequently than those receiving intravenous paracetamol. Reduced rescue analgesic requirement is clinically important because it minimizes opioid-related adverse effects and facilitates early postoperative recovery. Similar opioid-sparing effects of pre-emptive intravenous ibuprofen have been reported by Soyalp et al. [14].

Despite providing superior analgesia, intravenous ibuprofen was associated with a higher incidence of postoperative nausea and vomiting. Comparable findings have been reported in previous comparative studies evaluating intravenous ibuprofen and acetaminophen for perioperative pain management [10,12]. Importantly, no serious drug-related adverse events, including renal dysfunction, gastrointestinal bleeding, or cardiovascular complications, were observed in either treatment group, supporting the overall safety of both analgesic regimens in appropriately selected patients [6,10].

Both groups maintained stable intraoperative and postoperative haemodynamic parameters. However, patients receiving intravenous paracetamol demonstrated slightly more stable heart rate and mean arterial pressure throughout the observation period. Similar haemodynamic stability with intravenous paracetamol has been reported in comparative perioperative studies [6,10,12].

The present findings also support current opioid-sparing perioperative analgesic strategies. In addition to systemic non-opioid analgesics, regional analgesic techniques such as bilateral erector spinae plane block have demonstrated significant improvements in postoperative pain control and reductions in opioid consumption following laparoscopic cholecystectomy [15]. Furthermore, opioid-free anaesthesia has been shown to improve postoperative quality of recovery while maintaining satisfactory analgesia after laparoscopic cholecystectomy, highlighting the growing importance of multimodal analgesic approaches [16]. Therefore, the selection of intravenous paracetamol or intravenous ibuprofen should be individualized according to patient characteristics, including renal function, gastrointestinal bleeding risk, cardiovascular comorbidities, contraindications to non-steroidal anti-inflammatory drugs, and the anticipated inflammatory component of postoperative pain.

The strengths of this study include its randomized, double-blind design, standardized anaesthetic technique, uniform perioperative management, serial assessment of postoperative pain, and systematic evaluation of adverse events, all of which enhance the internal validity of the findings. This study has several limitations. It was conducted at a single tertiary-care centre with a relatively small sample size, which may limit the generalizability of the results. In addition, only the first 24 postoperative hours were evaluated, and long-term outcomes such as chronic postsurgical pain, quality of recovery, patient satisfaction, functional recovery, and cost-effectiveness were not assessed. Future multicentre randomized controlled trials with larger sample sizes and longer follow-up are warranted to further define the role of intravenous ibuprofen and intravenous paracetamol within multimodal analgesic protocols for laparoscopic cholecystectomy.

## Conclusions

Intravenous pre-emptive ibuprofen was found to be better than intravenous paracetamol for postoperative analgesia in patients undergoing elective laparoscopic cholecystectomy as evidenced by significantly lower postoperative Visual Analog Scale (VAS) pain scores. However, the increased analgesic efficacy was accompanied by a higher incidence of postoperative nausea and vomiting. The intravenous paracetamol had a more favourable safety profile, with more hemodynamic stability and less adverse effects. It was an appropriate alternative in patients for whom non-steroidal anti-inflammatory drugs are contraindicated or poorly tolerated. These data support a personalized approach to multimodal postoperative analgesia with choice of analgesic tailored to comorbidities, risk factors, and clinical priorities.
